# A DNA vaccine targeting VEE virus delivered by needle-free jet-injection protects macaques against aerosol challenge

**DOI:** 10.1038/s41541-022-00469-x

**Published:** 2022-04-22

**Authors:** John J. Suschak, Sandra L. Bixler, Catherine V. Badger, Kristin W. Spik, Steven A. Kwilas, Franco D. Rossi, Nancy Twenhafel, Melissa L. Adams, Charles J. Shoemaker, Erin Spiegel, Jay W. Hooper

**Affiliations:** 1 U.S. Army Medical Research Institute of Infectious Diseases, 1425 Porter St, Fort Detrick, MD 21702 USA; 2 PharmaJet, Inc., 400 Corporate Circle, Suite N, Golden, CO 80401 USA

**Keywords:** Alphaviruses, DNA vaccines

## Abstract

We have previously shown that DNA vaccines expressing codon optimized alphavirus envelope glycoprotein genes protect both mice and nonhuman primates from viral challenge when delivered by particle-mediated epidermal delivery (PMED) or intramuscular (IM) electroporation (EP). Another technology with fewer logistical drawbacks is disposable syringe jet injection (DSJI) devices developed by PharmaJet, Inc. These needle-free jet injection systems are spring-powered and capable of delivering vaccines either IM or into the dermis (ID). Here, we evaluated the immunogenicity of our Venezuelan equine encephalitis virus (VEEV) DNA vaccine delivered by either the IM- or ID-DSJI devices in nonhuman primates. The protective efficacy was assessed following aerosol challenge. We found that a prime and single boost by either the IM or ID route resulted in humoral and cellular immune responses that provided significant protection against disease and viremia. Although the ID route utilized one-fifth the DNA dose used in the IM route of vaccination, and the measured humoral and cellular immune responses trended lower, the level of protection was high and performed as well as the IM route for several clinical endpoints.

## Introduction

Venezuelan equine encephalitis virus (VEEV) is a mosquito-borne alphavirus that causes sporadic, but widespread, epidemics in North, Central, and South America^1^. Acute VEE disease is characterized by fever, headache, lymphopenia, myalgia, and malaise, with a mortality rate of less than 1%^2,3^. Multiple animal studies and documented laboratory accidents have proven that aerosolized VEEV is highly infectious with greater mortality rates than those observed in natural infection^4,5^. The increased clinical manifestations are hypothesized to be associated with an earlier and more severe infection in the central nervous system (CNS)^3^. Additionally, VEEV can be readily isolated from the environment, and easily grown to high titers^2^, resulting in VEEV being classified as a Category B priority pathogen with high potential for weaponization.

There are currently no FDA-approved VEEV vaccines for human use, but the U.S. Food and Drug Administration (FDA) has applied Investigational New Drug (IND) status to two vaccines intended to protect laboratory workers and at-risk personnel. TC-83 is a live-attenuated vaccine that can provide long-lasting immunity and protection from VEEV challenge, but approximately 20% of vaccinees do not develop a detectable immune response, and approximately 25% of vaccinees report adverse events^6,7^. C-84, a formalin-inactivated version of TC-83, is well-tolerated, but requires repeated boosting to maintain detectable neutralizing antibody responses in humans, and animal studies suggest C-84 provides suboptimal protection against aerosol viral challenge^8^. The limitations of these vaccines have prevented their licensure, necessitating the investigation of next-generation vaccine candidates^9–12^.

DNA vaccination has proven particularly effective at eliciting protective immune responses against alphavirus challenge^13–15^. We have previously shown that a candidate VEEV DNA vaccine (pWRG/VEE) expressing only the codon optimized envelope glycoprotein (GP) genes (E3-E2-6K-E1) delivered by intramuscular (IM) electroporation (EP) elicited robust antibody responses, including high-level VEEV neutralizing antibodies, in mice, rabbits, and nonhuman primates (NHPs)^16–18^. pWRG/VEE conferred protective immunity in mice and NHPs against aerosol VEEV challenge. Notably, pWRG/VEE vaccinated NHPs had significantly reduced fever responses compared to control animals, and serum viremia remained undetectable throughout the study^17^. A recent Phase I clinical trial provided further evidence that pWRG/VEE may confer protective immunity, as IM-EP and intradermal (ID)-EP delivery elicited anti-VEEV neutralizing antibodies in vaccinees^19^. These studies have demonstrated the effectiveness of EP, but logistical concerns remain. For example, unlike needle and syringe vaccines, electroporation devices require an electrical power source. In addition, many electroporation devices, such as the ones used in the Phase 1 trials, are not yet fully integrated and the control units are separate from the delivery devices.

A possible alternative for effectively delivering pWRG/VEE is jet injection. Jet injection has a successful history in vaccine delivery^20,21^. We have therefore begun investigating the protective efficacy of delivering pWRG/VEE by needle-free jet injection. PharmaJet, Inc. has developed needle-free jet injection technology that can deliver medications and vaccines using a narrow, precise fluid stream without the requirement for an external power source. A compressed spring that is manually reloaded using a reset station provides power for the devices. The PharmaJet Stratis injector delivers vaccines IM and subcutaneously (SC), while the PharmaJet Tropis injector delivers vaccine ID. Both devices have been granted U.S. FDA 510(k) clearance and are WHO prequalified. Both devices also show high acceptability among vaccinators, study subjects, and caregivers. For example, in an effort involving polio vaccination, acceptability survey data for Tropis indicated that 4638/4813 (94.7%) caregivers said they would be more likely to bring their child for vaccination in a future campaign that used jet injectors versus needle and syringe^22^. In a clinical study, the immunogenicity of an inactivated influenza vaccine delivered with the PharmaJet Stratis elicited antibody titers that were comparable to those measured following traditional needle and syringe delivery^23^. We have tested the immunogenicity of a bivalent hantavirus DNA vaccine delivered either IM or ID by PharmaJet devices in rabbits and NHPs^24^. NHP vaccination with both devices resulted in seroconversion, and a direct comparison showed that IM PharmaJet delivery yielded significant increases in neutralizing antibody titers when compared to needle and syringe delivery.

In this report, we evaluated the immunogenicity and protective efficacy of our pWRG/VEE DNA vaccine delivered by needle-free jet injection in NHPs against stringent aerosol challenge. Our results suggest that the PharmaJet Stratis and PharmaJet Tropis devices may provide a logistically simplified path forward for alphavirus DNA vaccines.

## Results

### pWRG/VEE DNA vaccine delivered by IM- or ID-DSJI elicits anti-VEEV antibodies in NHPs

**Fig. 1:**
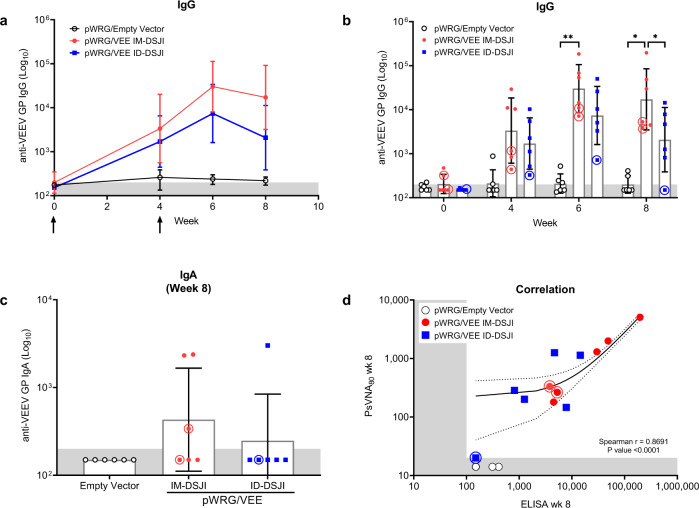
pWRG/VEE delivered by IM- or ID-DSJI elicits anti-VEEV GP binding antibodies. **a** Temporal anti-VEEV GP IgG responses and (**b**) total IgG antibody titers in vaccinated nonhuman primates (NHPs). **c** Total IgA antibody titers 4 weeks after the second vaccination in vaccinated NHPs. The shaded areas represent the assay limit based on the lowest sera dilution tested. Arrows represent vaccination time points. Data represent the group geometric mean ± geometric SD. *p < 0.05, **p < 0.01. **d** Correlation analysis of the VEEV ELISA and PsVNA_80_. For panels (**a**) and (**c**), *p* values were determined by one-way ANOVA with Tukey’s multiple comparison test. For panel (**b**), *p* values were determined by two-way ANOVA with Tukey’s multiple comparison. For panel (**d**), linear regression and 95% CI is shown as line and dotted lines, respectively. Circled symbols represent individual NHPs in VEEV-vaccinated groups that developed fever (defined as >3 SD above baseline lasting for more than 2 h) following VEEV aerosol challenge.

NHPs have been used extensively in VEEV aerosol challenge studies and are considered the most clinically relevant VEE animal model because they accurately reflect human disease^25^. Here, we evaluated the immunogenicity of pWRG/VEE delivered by disposable syringe jet injection (DSJI). Sera samples were obtained immediately before each vaccination and at weeks 6 and 8 for analysis of anti-VEEV GP IgG binding activity (Fig. 1). pWRG/empty vector vaccinated NHP controls did not seroconvert at any point following vaccination. Conversely, all pWRG/VEE vaccinated NHPs seroconverted following the first vaccination with either IM- or ID-DSJI. Anti-VEEV GP IgG titers were boosted following the second vaccination and IM delivery yielded a significant increase in IgG titers compared to ID delivery at week 8. Vaccination with both devices also generated antigen-specific IgA in a subset of NHPs by week 8, suggesting the potential for mucosal immunity (Fig. 1c).

**Fig. 2:**
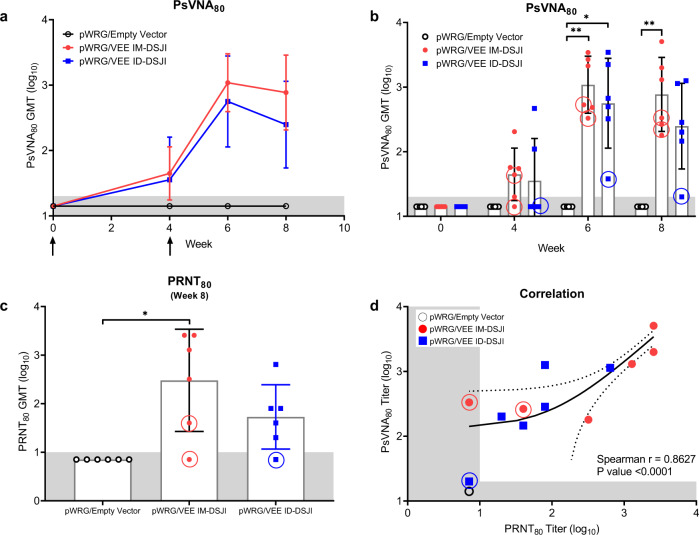
pWRG/VEE delivered by IM- or ID-DSJI elicits anti-VEEV neutralizing antibodies. **a** Temporal anti-VEEV GP neutralizing antibody responses and (**b**) PsVNA neutralizing antibody titers in vaccinated nonhuman primates. **c** PRNT titers 4 weeks post 2nd vaccination. The shaded areas represent the assay limit based on the lowest dilution of serum tested. Arrows represent vaccination time points. Data represent the group geometric mean ± geometric SD. *p < 0.05, **p < 0.01. **d** Correlation analysis to evaluate the relation of the VEEV PsVNA_80_ and PRNT_80_. For panels (**a**) and (**c**), *p* values were determined by one-way ANOVA with Tukey’s multiple comparison test. For panel (**b**), *p* values were determined by two-way ANOVA with Tukey’s multiple comparison. For panel (**d**), linear regression and 95% CI is shown as line and dotted lines, respectively. Circled symbols represent individual animals in VEEV-vaccinated groups that developed a fever >3 °C above baseline after VEEV aerosol challenge.

pWRG/VEE vaccination by both delivery methods also elicited high levels of anti-VEEV neutralizing antibodies as measured by PsVNA. As with binding antibody titer, neutralizing capability was boosted by the second vaccination regardless of device or vaccination route (Fig. 2a, b). A comparison of the week 6 and week 8 neutralizing antibody titers between the IM-DSJI and ID-DSJI groups demonstrated neutralizing antibody titers that trended higher in IM-DSJI vaccinated NHPs, but the difference did not reach statistical significance. To confirm the PsVNA results, we also quantified week 8 neutralizing antibodies by the traditional PRNT assay (Fig. 2c). PRNT_80_ titers were strongly correlated (Spearman *r* = 0.8627, *p* > 0.0001) with those measured by PsVNA_80_ (Fig. 2d). Likewise, the week 8 PsVNA_80_ titers were strongly correlated (Spearman *r* = 0.8691, *p* > 0.0001) with the ELISA data (Fig. 1d).

### IM-DSJI, but not ID-DSJI, delivery of pWRG/VEE yields antigen-specific cellular immune responses in NHPs

**Fig. 3:**
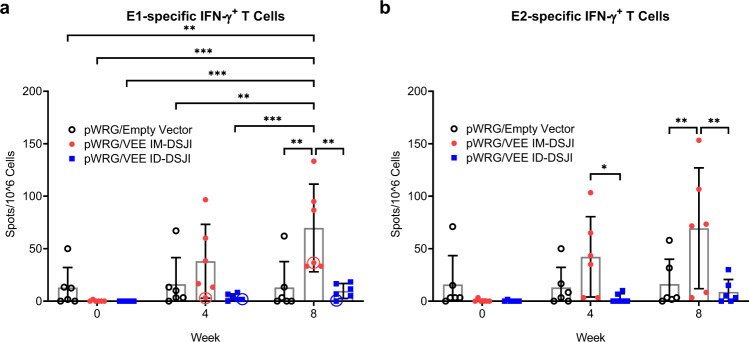
pWRG/VEE delivered by IM-, but not ID-, DSJI elicits anti anti-VEEV GP cellular immunity. **a** VEEV E1- and (**b**) E2-specific IFN-γ^+^ T cells. Peripheral blood mononuclear cells were stimulated with pools of 15-mer, overlapping peptides spanning the VEEV E1 or E2 proteins. Data represent the group mean ± SD. Statistical analysis was performed using a two-way ANOVA with Tukey’s multiple comparison test. *p < 0.05, **p < 0.01, ***p < 0.001. Circled symbols represent individual animals in VEEV-vaccinated groups that developed a fever >3 °C above baseline after VEEV aerosol challenge.

IM injection of pWRG/VEE, whether by needle and syringe or EP, elicits high levels of anti-VEEV E1- and E2 T cells in mice^13,14^. We, therefore, quantified the VEEV GP-specific IFN-γ^+^ T cell populations in the DSJI-vaccinated NHPs (Fig. 3a, b). As with the humoral response, pWRG/empty vector vaccinated animals did not generate a significant anti-VEEV E1- or E2-specific T cell response. IM-DSJI delivery of pWRG/VEE elicited a potent T cell response, with significant increases following the second vaccination. Interestingly, ID-DSJI delivery did not elicit high numbers of anti-VEEV E1- or E2 T cells, instead exhibiting anti-VEEV E1- and E2 T cell numbers that were statistically equivalent to pWRG/empty vector vaccinated NHPs.

### Protective efficacy of pWRG/VEE delivered by IM- or ID-DSJI against aerosol VEEV challenge in NHPs

Eight weeks after the second vaccination, all NHPs were challenged with a target dose of 1 × 10^8^ PFU of VEEV by the aerosol route. The average inhaled dose was calculated to be 6.43 × 10^7^ PFU by neutral red plaque assay (Supplementary Table 1). All NHPs were monitored for development of clinical disease signs by veterinary staff blinded to study groups. No animals displayed overt disease signs during subjective daily observations, although several NHPs in the pWRG/empty vector group turned their back toward the observer. These observations were consistent with some of the behaviors noted in previous studies^3,17^.

**Fig. 4:**
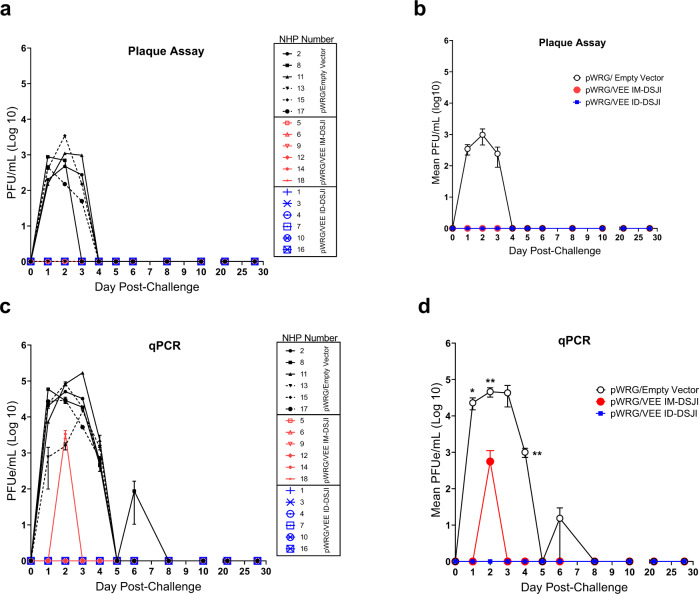
Viremia following VEEV aerosol exposure. **a** Individual nonhuman primate (NHP) serum VEEV plaque assay data. **b** Group plaque assay data. Mean ± SEM are plotted for each group. **c** Individual NHP RT-qPCR determination of VEEV viral load present in plasma. Viral load is expressed in equivalent (e) PFU/mL and was interpolated from a standard curve generated from RNA extracted from challenge virus. Samples were analyzed in technical triplicate. Error bars reflect standard deviation. The lower limit of quantitation for the qPCR assay is 100 PFU/mL. **d** Group qPCR data. Mean ± SEM are plotted. For panel (**d**), *p* values were determined by one-way ANOVA with Tukey’s multiple comparison test. *p < 0.05, **p < 0.01.

Infectious virus in the sera was measured by plaque assay, while qPCR was used to detect viral nucleic acid in plasma. All pWRG/VEE vaccinated NHPs, regardless of device used, remained aviremic following challenge as measured by plaque assay (Fig. 4a, b). Conversely, infectious virus was detected in five of the six pWRG/empty vector vaccinated NHPs on day 1 post-challenge. The exception was NHP #13. Viremia peaked on day 2 post-challenge, with a mean group titer of 980 PFU/mL and remained detectable until day 4 post-challenge. The qPCR data largely mirrored the results of the plaque assay. pWRG/empty vector vaccinated NHPs had significantly higher viral loads than both pWRG/VEE vaccinated groups on days 1, 2, and 4 post-challenge (Fig. 4c, d). The pWRG/empty vector vaccinated NHP (#13) that was negative for infectious virus did have detectable levels of viral genome on day 2 post-challenge. However, viral genome in this animal was nearly a log lower than the other animals in that group and was undetectable by day 3 post-challenge. One IM-DSJI-vaccinated NHP (#12) had a low level of viral genome (3350 pfu/mL) detected on day 2 post-challenge, while all ID-DSJI-vaccinated animals were negative by qPCR. Anti-VEEV neutralizing antibodies were detected in all NHPs vaccinated with the pWRG/empty vector at the terminal time point (4 weeks post-challenge), confirming that all six NHPs in that group were successfully infected following aerosol challenge (Supplementary Fig. 1).

**Fig. 5:**
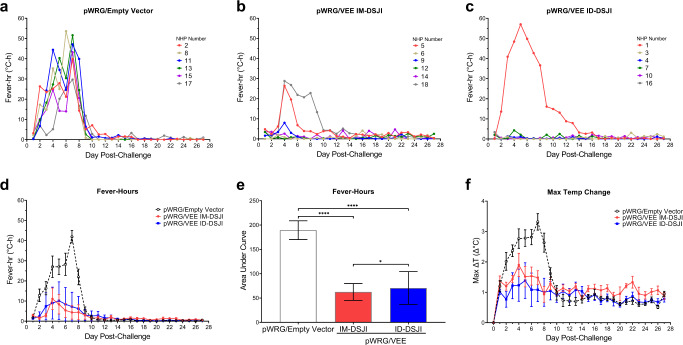
Fever responses in nonhuman primates (NHPs) following VEEV aerosol challenge. Fever-hours for individual animals in the (**a**) pWRG/empty vector (**b**) pWRG/VEE IM-DSJI and (**c**) pWRG/VEE ID-DSJI groups. Fever-hours are the sum of the significant temperature elevations (defined as >3 SD above baseline) in a 24 h period. The group means ± SEM and area under the curve (AUC) are presented in (**d**) and (**e**), respectively. **f** Maximum temperature elevation above baseline in a 24 h period. Data are presented as mean ± SEM. *p* values were determined using a one-way ANOVA with Tukey’s multiple comparison test. *p < 0.05, ****p < 0.0001.

Since fever is a key hallmark of VEE disease, we continuously monitored all NHPs by M00 telemetry implants. All pWRG/empty vector vaccinated NHPs (six of six) developed sustained fever, with four of the six developing hyperpyrexia (Fig. 5a, d, f). Five of the six pWRG/VEE IM-DSJI vaccinated NHPs exhibited short (lasting less than 2 h) and isolated fever episodes throughout the post-challenge period, but only two of these NHPs (#5 and #18) exhibited sustained fever responses that lasted four or more consecutive days (Fig. 5b, d). Only one NHP (#18) in this group exhibited a fever response commensurate with the peak responses measured in the pWRG/empty vector group. Notably, only one of the six NHPs in the pWRG/VEE ID-DSJI group developed fever (Fig. 5c, d). This NHP (#1) developed sustained fever and hyperpyrexia and had the lowest anti-VEEV GP humoral response as measured by both ELISA and PsVNA (Figs. 1 and 2). Area under the curve analysis of grouped fever-hour data showed that pWRG/VEE vaccinated NHPs had significantly fewer fever-hours than animals receiving pWRG/empty vector (Fig. 5e). Additionally, the pWRG/VEE IM-DSJI group exhibited fewer fever-hours than the pWRG/VEE ID-DSJI group, however, this can be attributed to the sustained fever of NHP #1 in the latter. Although activity was also monitored by the telemetry implants, no significant differences were observed between the control and vaccinated groups.

**Fig. 6:**
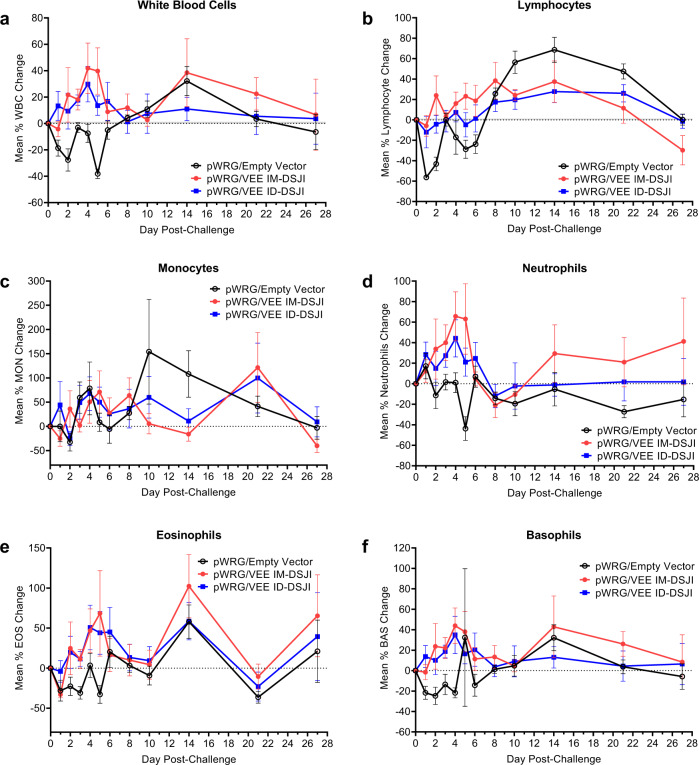
VEEV DNA vaccine delivered by IM- or ID-DSJI prevents decline in white blood cell populations following aerosol challenge. Percent change from baseline in (**a**) white blood cells, (**b**) circulating lymphocyte populations, (**c**) circulating monocyte populations, (**d**) circulating neutrophil populations, (**e**) circulating eosinophil populations, and (**f**) circulating basophil populations following aerosol VEEV exposure. The dashed line represents the baseline values as determined by the average of 3 independent measurements on days −7, −5, and −3 relative to challenge. Data represent the group mean ± SEM. Group and pairwise statistical analysis is presented in Supplementary Table 3.

Lymphopenia has been reported to be a biomarker of VEE disease in NHPs^26^. To monitor lymphopenia and other potential changes in white blood cell populations, complete blood counts were measured post-VEEV challenge and compared to average baseline counts measured on multiple days pre-challenge. All pWRG/empty vector vaccinated NHPs (six of six) developed leukopenia as well as lymphopenia, with an average decrease of 56.3% in peripheral lymphocytes from baseline within 24 h of VEEV infection (Fig. 6). Peripheral lymphocyte populations briefly recovered on day 3 post-challenge but decreased again with an average reduction of 28.7% from baseline by day 5 post-challenge. This pattern is characteristic of the biphasic immune response to VEEV infection as virus spreads from the periphery into the CNS^27^. In contrast, IM-DSJI-vaccinated animals largely maintained consistent levels of circulating lymphocytes, which were significantly different from the pWRG/empty vector NHPs on days 1, 2, and 5 post-challenge (Supplementary Table 3). Similarly, five of the six ID-DSJI-vaccinated animals maintained lymphocyte counts 24 h post-challenge (average 12.1% decrease), with significant differences from the pWRG/empty vector vaccinated animals on day 2 post-challenge. One NHP (#1) developed lymphopenia, with a maximum decrease of 62.2% on day 5 post-challenge in lymphocyte populations suggesting that the animal had developed disease (Supplementary Fig. 2). Marked neutropenia was also observed in the pWRG/empty vector vaccinated NHPs on day 5 post-challenge, which was statistically significant compared to both the IM-DSJI and ID-DSJI groups. A similar overall trend was observed for eosinophil and basophil counts. While transient changes in monocytes occurred in all three groups, there was not a significant difference between the pWRG/empty vector group and the IM-DSJI and ID-DSJI animals.

### pWRG/VEE delivered by DSJI limits development of encephalitis in the brain of NHPs

**Fig. 7:**
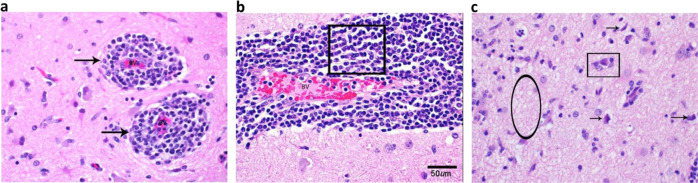
Encephalitis typical of VEEV infection in nonhuman primates (NHPs). Representative pathology in the cerebrum of NHPs challenged with VEEV by aerosol. **a** NHP #17 (pWRG/empty vector), Cerebrum. Virchow-Robbins spaces (arrows) surrounding blood vessels (BV) in the brain. This area is moderately expanded by inflammatory cells. H&E 20X. **b** NHP #8 (pWRG/empty vector), Cerebrum. Virchow-Robbin space is moderately expanded by lymphocytes and macrophages (boxed area), or nonsuppurative inflammation. This is a key feature in differentiating VEEV from Eastern and Western equine encephalitis viruses (EEEV and WEEV, respectively). H&E 20X. **c** NHP #18 (IM-DSJI), Cerebrum. There are degenerating and dead neurons (arrows) characterized by shrunken cells that are angular and hypereosinophilic (dark red/pink) with condensed nuclei. There is satellitosis (rectangle) characterized by glial cells surrounding the neuron, like satellites. Spongiosis (oval) is present in this section as the neuropil is lost or edematous giving the look of a sponge. H&E 40X.

At the conclusion of the study, all animals were necropsied and examined for signs of encephalitis. None of the animals exhibited gross lesions that were attributable to VEEV challenge. Histologically, the most notable finding was evidence of encephalitis in 11 of the 18 animals, with disease being distributed multifocally in areas of the cerebrum, particularly the corpus striatum and thalamus (Table 1). Notably, three of the six animals in the IM-DSJI group and four of the six animals in the ID-DSJI group did not show any evidence of encephalitis. Animals were considered to have encephalitis if two or more of the following histologic changes were present: nonsuppurative inflammation expanding the Virchow-Robin space, neuronal degeneration and necrosis, gliosis, satellitosis, and spongiosis (Fig. 7). The severity of the histological lesions in the brains of NHPs in this study correlated with the telemetry data as the pWRG/VEE vaccinated animals that developed sustained fever (IM-DSJI: #5 and #18 and ID-DSJI: #1) also had histologic evidence of encephalitis in the brain. The highest encephalitis scores for each group were NHP #8 in the pWRG/empty vector group (score of 11), NHP #18 in the IM-DSJI group (score of 20), and NHP #1 in the ID-DSJI group (score of 13). Histopathology performed on vaccination site and tracheobronchial lymph node tissue produced no significant findings. Viral antigen could not be detected by immunohistochemistry in any animal, suggesting that virus was cleared in all animals by the terminal endpoint (4 weeks post-challenge).

**Table 1:** Pathology scores of isolated brain sections.

Vaccine	NHP number	Frontal cortex	Corpus striatum	Thalamus	Mesencephalon	Cerebellum	Pons	Medulla oblongata	Meningitis	Total score
Empty vector	2	0	1	1	0	0	0	0	0	2
Empty vector	8	2	2	2	2	0	2	0	1	11
Empty vector	11	1	1	1	0	0	0	0	1	4
Empty vector	13	0	1	1	1	0	0	0	0	3
Empty vector	15	1	1	1	0	0	0	0	1	4
Empty vector	17	1	1	1	0	0	0	0	0	3
Empty vector	Total Score	5	7	7	3	0	2	0	3	27
IM-DSJI	5	1	2	2	0	0	0	0	1	6
IM-DSJI	6	0	0	0	0	0	0	0	0	0
IM-DSJI	9	0	1	1	0	0	0	0	0	2
IM-DSJI	12	0	0	0	0	0	0	0	0	0
IM-DSJI	14	0	0	0	0	0	0	0	0	0
IM-DSJI	18	3	3	3	3	0	3	3	2	20
IM-DSJI	Total Score	4	6	6	3	0	3	3	3	28
ID-DSJI	1	2	2	1	2	0	2	2	2	13
ID-DSJI	3	0	0	0	0	0	0	0	0	0
ID-DSJI	4	0	1	0	0	0	0	0	1	2
ID-DSJI	7	0	0	0	0	0	0	0	0	0
ID-DSJI	10	0	0	0	0	0	0	0	0	0
ID-DSJI	16	0	0	0	0	0	0	0	0	0
ID-DSJI	Total Score	2	3	1	2	0	2	2	2	15
All Animals	Total Score	11	16	14	8	0	7	5	9	70

Pathology score based on percentage of tissue affected by the histologic changes 0-negative, 1-minimal, >0–10%, 2-mild, >10–25%, 3-moderate, >25–50%, and 4-marked, >50–75%.

*NHP* Nonhuman primate.

## Discussion

We have previously shown that EP delivery of pWRG/VEE protects NHPs from VEE disease and is immunogenic in a Phase I clinical trial^19^. As a possible pre-cursor to clinical trials, the objective of this study was to determine if needle-free jet injection of our pWRG/VEE vaccine could protect NHPs from aerosol VEEV challenge. Although needle-free delivery strategies have been undertaken for other viruses, this is the first report, to our knowledge, examining this approach for a VEEV DNA vaccine. We also believe it to be the first report to quantify vaccine-elicited anti-VEEV E1- and E2-specific T cell response in NHPs.

Anti-VEEV GP neutralizing antibodies are considered the strongest correlate of protection against VEEV aerosol challenge^17,28,29^. Vaccination by both IM-DSJI and ID-DSJI elicited high levels of VEEV GP-specific antibodies following the second vaccination, and neutralizing antibody levels generated by DSJI reached similar levels to those measured in our previous IM-EP studies^17^. The critical role anti-VEEV GP antibodies play in protection from VEEV challenge was illustrated by the development of sustained fever and lymphopenia in NHP #1. This NHP consistently demonstrated the weakest humoral response of all pWRG/VEE vaccinated animals and was the only pWRG/VEE vaccinated NHP to develop disease symptoms commensurate to pWRG/empty vector controls. It is unclear why NHP #1 failed to generate high-level immunity, but the lack of detectable viremia in this animal suggests that even a low level of neutralizing antibodies can confer some level of protection from aerosol challenge.

While vaccination with pWRG/VEE elicited protective immunity regardless of the delivery route, we were able to quantify differences in the magnitude of response generated by IM-DSJI and ID-DSJI vaccination. IM-DSJI vaccinated NHPs exhibited higher anti-VEEV GP IgG and neutralizing antibody titers than did ID-DSJI. Similar results have been described in other studies comparing IM and ID delivery of DNA vaccines. ID-EP vaccinees in our Phase I clinical trial exhibited an approximately two-fold reduction in anti-VEEV neutralizing antibody titers compared to IM-EP vaccinated individuals^19^. This effect appeared to be at least partially dose-dependent. The increased humoral immunogenicity of IM delivery in the current study may be of a similar nature because IM-DSJI vaccinated animals received five times the amount of pWRG/VEE DNA as ID-DSJI vaccinated animals. Possibly compounding this dose-dependent effect is the nature of NHP skin. In our previous NHP PharmaJet study, we showed that vaccination with a prototype IM device yielded higher neutralizing antibody titers than did a prototype ID device in a Sin Nombre virus (SNV) NHP model^24^. That report attributed the disparity in neutralizing antibody titers to the difficulty of penetrating NHP skin because ID vaccinated NHPs had reduced bleb size and fluid remaining on the skin. Despite that finding, needle-free ID jet injection proved highly immunogenic in both studies and clearly protected NHPs from VEE disease in this study. Future NHP studies may investigate how each delivery method shapes the humoral response and whether increasing the ID dose can improve neutralizing antibody levels.

Perhaps the most striking difference in the immune profile was the near absence of antigen-specific IFN-γ^+^ T cells in ID-DSJI vaccinated NHPs, although these data agree with a previous report suggesting that robust anti-VEEV T cell responses are difficult to detect even after VEEV infection^30^. Why IM-DSJI delivery resulted in quantifiable T cell numbers remains unanswered, but antigen dose can have a positive effect on T cell proliferation and memory^31^. We have not previously attempted to measure anti-VEEV GP T cell responses in NHPs, so it is possible that other IM delivery methods have a similar effect on cellular immunity. The role of cellular immunity in protection from VEEV aerosol challenge remains unclear, but data suggest that T cells can control VEEV infection in the CNS of mice^32,33^. Regardless, the low levels of peripheral VEEV GP-specific T cells did not appear to negatively impact the overall vaccine efficacy as only one ID-DSJI vaccinated NHP developed fever and lymphopenia following aerosol challenge. It is possible that the reduced T cell response contributed to the increased frequency of low level brain pathology in ID-DSJI vaccinated NHP (Table 1). These data would further suggest that neutralizing antibodies are the primary correlate of protection from VEEV aerosol infection. Further investigation is required to determine if IM delivery provides an inherent advantage in driving anti-VEEV T cell immunity, as well as how cellular immunity mediates protection from VEEV aerosol challenge in NHPs.

Despite the increased immunogenicity measured in IM-DSJI vaccinated NHPs, it appears that ID-DSJI delivery provided, at a minimum, comparable protection from aerosol VEEV challenge. None of the vaccinated animals had detectable viremia by plaque assay following aerosol challenge, with only one IM-DSJI animal positive by PCR at a single time point. Moreover, only one animal in the ID-DSJI group developed a fever and showed signs of sustained encephalitis at the termination of the study. Although the underlying mechanisms governing this effect were not exhaustively investigated here, multiple possibilities for the similar levels of protective efficacy by ID-DSJI delivery exist. First, ID DNA vaccination can result in improved antibody avidity compared to IM delivery^34^. Such an effect would provide for rapid clearance of antigen from the host, and at least partially explain the lack of disease observed in ID-DSJI vaccinated animals. However, improved antibody avidity is unlikely as IM-DSJI delivery yielded neutralizing antibody titers that trended higher. Perhaps a more likely possibility is the capability of ID delivery to elicit protective mucosal immunity^35–37^. It is feasible that ID-DSJI vaccination augmented the mucosal immune response, significantly limiting the ability of the virus to replicate there and then spread quickly into the CNS. While we did not detect high levels of VEEV GP-specific IgA in the periphery, we did not attempt to quantify mucosal IgA from nasal passages or bronchoalveolar lavage (BAL), as this may have caused significant damage to the respiratory epithelium prior to aerosol challenge. Future studies examining these immune compartments following IM-DSJI and/or ID-DSJI vaccination may provide a clearer understanding of the delivery route on mucosal immunity.

In summary, we show that a simplified DNA vaccine delivery system provides protection from aerosol VEEV challenge. DNA vaccination with either the PharmaJet IM-DSJI or ID-DSJI delivery system elicits robust immune responses following two vaccinations with pWRG/VEE in an NHP model. Our current findings are consistent with previous IM-EP studies demonstrating that a DNA vaccine expressing the E1 and E2 glycoproteins of VEEV elicits high levels of antigen-specific neutralizing antibodies. We also show for the first time that vaccination with pWRG/VEE elicits IFN-γ^+^ T cells in NHP. These results suggest that PharmaJet delivery of pWRG/VEE may be an effective method for generating protective immunity in humans. Determining the optimal device (i.e., Stratis or Tropis) for VEE vaccine in humans will need to take into consideration not only the immune performance, but also dosing volume limitations and other subtle differences in the IM and ID devices that can impact the cost and utility of the final drug product.

## Methods

### Ethics statement

Research was conducted under an animal protocol approved by the IACUC at the United States Army Medical Research Institute of Infectious Diseases (USAMRIID). This protocol covered all experiments performed during this study, including the method of euthanasia, and complied with the Animal Welfare Act, PHS Policy, and other Federal statutes and regulations relating to animals and experiments involving animals. The facility where this research was conducted is accredited by the Association for Assessment and Accreditation of Laboratory Animal Care International and adheres to principles stated in the Guide for the Care and Use of Laboratory Animals, National Research Council, 2011. Animals were housed one per cage in stainless steel cages with squeeze capabilities for handling in Animal Biosafety Level-3 (ABSL-3) biocontainment. Animal rooms were controlled for temperature and humidity and maintained on a 12 h light/12 h dark cycle. Appropriate enrichment was provided in the form of toys, nutritional items, and mirrors. Animals were provided 2050 C Teklad Monkey Chow (Harlan Laboratories, Indianapolis, IN), water *ad libitum* via an automatic watering system, and hydrating fruits, liquids, and other food items, as determined appropriate by the investigators or USAMRIID Veterinary Medicine Division staff. Animals were anesthetized prior to all handling and/or manipulations (including physical examinations, aerosol challenge, vaccination, and blood collections) with ketamine (10 mg/kg; Henry Schein, Melville, NY) or Telazol^®^ (3 mg/kg; Zoetis, Parsippany, NJ). Animals were euthanized at the end of study by intracardiac administration of a pentobarbital-based euthanasia solution (0.3–0.4 mL/kg) under deep anesthesia with Telazol (7 mg/kg; Zoetis).

### DNA vaccines

Construction of the pWRG/VEE DNA vaccine candidate expressing the E3-E2-6K-E1 genes of VEEV subtype IAB was previously described^17^. Briefly, codon optimization of the structural genes, minus the capsid protein coding region, of VEEV IAB strain Trinidad donkey (TrD) (Genbank accession number L01442) was accomplished using the Gene Optimizer bioinformatic algorithm followed by synthesis of the codon optimized genes (Geneart, Regensburg, Germany). pWRG/VEE was constructed by cloning the synthesized codon optimized genes into the NotI and BglII restriction sites of pWRG7077. Research-grade plasmids were manufactured by Aldevron (Fargo, ND). Prior to vaccination, the DNA stocks were thawed at ambient temperature and inverted to ensure mixing. The DNA stocks were diluted in sterile 1X phosphate-buffered saline (PBS) (Gibco, Gaithersburg, MD) without magnesium or calcium to achieve the necessary concentrations for vaccination.

### Vaccinations

A total of 18 healthy adult male and female cynomolgus macaques (>4.0 kg) were obtained from the NHP colony at USAMRIID. All animals were confirmed negative for simian immunodeficiency virus (SIV), simian T-lymphotropic virus (STLV), herpes B virus, filovirus, and simian retrovirus (SRV) types 1, 2, 3, and 5. Animals were also screened for the presence of serum neutralizing antibodies against VEEV IAB by pseudovirion neutralization assay (PsVNA) prior to study assignment as described below. Macaques were then randomized to three vaccination groups (*n* = 6) balanced by sex and weight. Animals were vaccinated twice at weeks 0 and 4 with either pWRG/empty vector or pWRG/VEE. One group received 2.0 mg pWRG/empty vector by PharmaJet (Golden, CO) Stratis (IM-DSJI) in a volume of 0.5 mL and 0.4 mg pWRG/empty vector by PharmaJet Tropis (ID-DSJI) in a volume of 0.1 mL. One group received 2.0 mg pWRG/VEE by IM-DSJI in a volume of 0.5 mL. One group received 0.4 mg pWRG/VEE by ID-DSJI in 0.1 mL.

Prior to vaccination, macaques were anesthetized with an IM injection of ketamine (10 mg/kg) to minimize startle reflex and ensure immobilization for accurate administration of the vaccines. The hair covering the skin of the triceps muscle (IM-DSJI) and/or the hair overlying the posterior portion of the axilla (ID-DSJI) was removed using clippers and the site of vaccination was disinfected prior to vaccination. The device was placed against the skin at the site of vaccination and injection was achieved by pressing a button on the device.

During the vaccination phase, periodic blood collection was performed under anesthesia for the assessment of immune responses to vaccination. Serum was isolated from all animals at weeks 0, 4, 6, and 8. Peripheral blood mononuclear cells (PBMC) were isolated at weeks 0, 4, and 8. A summary of key activities during the vaccinated phase in provided in Supplementary Table 2.

### ELISA

High binding ELISA plates (Corning, Corning, NY) were coated with virus-like particles (VLP) expressing the VEEV E1/E2 antigen encoded within the DNA vaccine. VLP were produced by co-transfection of pWRG/VEE and a scaffolding Gag protein^13^. For the construction of the Gag-encoding plasmid, the first 538 residues of murine leukemia virus (MLV) Gag-Pol ORF (GenBank: AF033811.1) were codon optimized, synthesized, and cloned into pWRG7077 using flanking 5’ NotI and 3’ BglII restriction sites relative to the transgene insert (Atum Inc, Menlo Park, CA). HEK293T cells were seeded in T150 flasks (Corning) prior to transfection with 27 µg of pWRG7077-Gag and 9 µg of pWRG7077-VEEV plasmid DNA using Fugene 6 (Roche, Indianapolis, IN) according to manufacturer’s instructions. Cell supernatants were collected at 24 and 48 h post-transfection, pooled, clarified by centrifugation, and filtered through a 0.45 µm filter. VLPs were concentrated through a Centricon^®^ filter unit with a 100 kDa cutoff (EMD Millipore, Burlington, MA, USA) according to manufacturer’s instructions. VLP were then pelleted through a 20% sucrose cushion in virus resuspension buffer (VRB; 130 mM NaCl, 20 mM HEPES, pH 7.4) by centrifugation for 2 h at 106,750 × g in an SW32 rotor at 4 °C. VLP pellets were resuspended overnight in VRB at 4 °C, pooled, and diluted ten-fold with VRB. The diluted VLPs were re-pelleted without a sucrose cushion as described above. VLPs were resuspended in 1/1000 volume of VRB relative to starting supernatant and then stored at −80 °C. ELISA plates were coated at a concentration of 200 ng/well, and then incubated overnight at 4 °C. The following day, plates were washed with PBS containing 0.2% Tween-20 and then blocked with Neptune Block (ImmunoChemistry Technologies, Bloomington, MN) for 2 h at 37 °C. Plates were washed again, prior to being loaded with two-fold serial dilutions of NHP sera in duplicate (dilution range 1:200 to 1:409,600). Serum dilutions were carried out in Neptune Block. Plates were incubated at 37 °C for 1 h prior to being washed, and then incubated with a 1:1000 dilution of horseradish peroxidase (HRP) conjugated goat anti-monkey (SeraCare Life Sciences, Gaithersburg, MD; IgG – catalog no. 5210-0159; IgA – catalog no. 5210-0154) in Neptune Block for 1 h at 37 °C. Plates were washed again, and then developed with SureBlue TMB substrate (SeraCare Life Sciences). Absorbance at the 450 nm wavelength was detected with a Tecan M1000 microplate reader (Tecan Group Ltd, Switzerland). Pooled naïve sera collected prior to vaccination were used as an internal control for each assay group. A plate cutoff value was determined based on 2.5 x average absorbance + 2.5 x SD of the pooled pre-vaccination sera from each respective group. End-point titers were determined using GraphPad Prism 9 (GraphPad Software, La Jolla, CA).

### Pseudovirion neutralization assay

The pseudovirion neutralization assay (PsVNA) for detection of neutralizing antibodies in sera uses a replication-restricted, recombinant vesicular stomatitis virus (rVSV*ΔG) expressing luciferase, which is pseudotyped with the VEEV IAB E1/E2 glycoproteins (TrD)^38^. Briefly, heat-inactivated NHP sera (56 °C for 30 min) was first diluted 1:20, followed by five-fold serial dilutions that were mixed with an equal volume of Eagle’s minimum essential medium with Earle’s salts and 10% FBS containing 4000 fluorescent focus units of VEEV E1/E2 pseudovirions. This mixture was incubated overnight at 4 °C. Following this incubation, 50 μL was inoculated onto Vero 76 (ATCC, Manassas, VA) cell monolayers in a clear bottom, black-walled 96-well plate in duplicate. Plates were incubated at 37 °C for 18–24 h. The media was discarded and cells were lysed according to the luciferase kit protocol (Promega, Madison, WI). A Tecan M200 Pro (Tecan Group Ltd) was used to acquire luciferase data. The values were graphed using GraphPad Prism 9 software and used to calculate the percent neutralization normalized to cells alone and pseudovirions alone as the minimum and maximum signals, respectively. The percent neutralization values for duplicate serial dilutions were plotted. Eighty percent PsVNA (PsVNA_80_) titers were interpolated from 4-parameter curves, and geometric mean titers were calculated.

### Plaque reduction neutralization test (PRNT)

PRNT were conducted on Vero 76 (ATCC) cells. Ten-fold serial dilutions of heat-inactivated sera were prepared in 1X Hanks Balanced Salt Solution (HBSS) without HEPES, with phenol red (Corning) supplemented with 2% heat-inactivated fetal bovine serum (HI-FBS) (Hyclone, Logan, UT), 2% HEPES (Sigma, Burlington, MA), and 1% penicillin/streptomycin (Gibco). Serum dilutions were mixed with 100 PFU of VEEV and incubated overnight at 4 °C. The serum/virus suspension was transferred to monolayers of Vero 76 (ATCC) cells in six-well cell culture plates (Corning) in duplicate and incubated for 1 h at 37 °C. The monolayers were overlaid with 2 mL of primary overlay media containing 2X EBME (USAMRIID) with HEPES (Gibco), HI-FBS (Hyclone), L-glutamine (Gibco), penicillin/streptomycin (Gibco), MEM non-essential amino acid (NEAA) solution (Gibco), gentamicin (Sigma), and 1% agarose (GeneMate), and incubated for 24–30 h at 37 °C. The cells were overlaid with 2 mL of primary overlay media supplemented with neutral red (USAMRIID). Plaques were counted 18–26 h later and the titer of the test sera was expressed as the reciprocal of the greatest serum dilution that neutralized eighty percent of the virus in media+virus control wells.

### ELISPOT assay

NHP T cell ELISPOT reagents were obtained from Mabtech (Cincinnati, OH). VEEV E1 and VEEV E2 specific IFN-γ^+^ T cells were quantified per manufacturer instructions. Positive control wells were stimulated with a 1:1000 dilution of anti-CD3 monoclonal antibody CD3-1 (Mabtech kit catalog no. 3421M-4HST-10). Pooled 15-me peptides with an 11-base overlap spanning the VEEV IAB E1 or E2 envelope glycoprotein (Pepscan, Lelystad, Netherlands) were added to the appropriate wells at 10 µg/mL. Cells were incubated in RPMI media (Gibco) containing 10% HI-FBS (Hyclone) and 5% penicillin and streptomycin (Gibco) for 20 h at 37 °C in 5% CO_2_. Positive spots were visualized on a CTL Imager (Cellular Technology Ltd., Shaker Heights, OH) and counting was performed with Immunospot software (CTL).

### Aerosol challenge of cynomolgus macaques

Animals were acclimated in ABSL-3 animal rooms for 8 days prior to challenge. Before aerosol exposure, macaques were anesthetized by IM injection of 4 mg/kg Telazol (Zoetis) and a whole-body plethysmograph was taken for 3 min to determine the respiratory capacity of each animal. NHPs were then inserted into a class III biological safety cabinet located inside a Biosafety Level 3 containment suite and exposed in a head-only aerosol chamber to a VEEV aerosol created by a three-jet Collison nebulizer (BGI, Inc., Waltham, MA) for 10 min as previously described^17,18^. A stock of VEEV IAB strain TrD was diluted to a starting concentration of 2.15 × 10^10^ plaque forming units (pfu)/mL in 1X HBSS (Corning) containing 1% HI-FBS (Hyclone) for use in aerosol generation. The VEEV IAB strain TrD, provided by the Glass laboratory at USAMRIID, was originally isolated in 1943 from the brain of a donkey on Trinidad. Sucrose-purified VEEV IAB TrD was obtained from supernatants of infected baby hamster kidney (BHK) cells, pooled, and filtered through a 0.2 µm filter. The stock titer as determined by plaque assay was 2.05 × 10^12^ pfu/mL. The identity of the VEEV IAB TrD stock was confirmed by PCR and sequencing, with no contamination detected. The inhaled dose for each NHP was determined by plaque assay of samples collected from the all-glass impinger (AGI) attached to the aerosol chamber at the time of challenge as described above.

Following challenge, the NHPs were monitored at least once daily for clinical signs of illness for a period of 28 days, including neurological signs, changes in activity and behavior, and responses to stimuli using predetermined criteria. Veterinary staff performing animal observations were blinded to group designations. Anesthetized physical examinations and blood collection were also performed on days 7, 5, and 3 prior to challenge and days 1, 2, 3, 4, 5, 6, 8, 10, 14, 21, and 27 post-challenge. At the designated end of study (28 days post-challenge) all animals were humanely euthanized under deep anesthesia via intracardiac administration of a pentobarbital-based euthanasia solution.

### Challenge phase assays

Complete blood counts were performed on EDTA-treated whole blood using a VETSCAN HM5 Hematology Analyzer instrument (Abaxis Inc., Union City, CA) and multi-species software. Serum viremia was quantified by plaque assay as described above for inhaled dose determination.

For determination of viral loads by quantitative RT-PCR, plasma samples were inactivated by mixing with TRIzol™ LS (Ambion, Austin, TX) at a ratio of 3:1 and were then frozen at −80 °C until subsequent extraction. RNA for RT-qPCR analysis was extracted with the Qiagen EZ1 Advanced robot with the EZ1 Virus Mini 2.0 Kit (Qiagen, Germantown, MD) according to the manufacturer’s directions. Standard curves were made from serial 10-fold dilutions of RNA generated from a stock of VEEV IAB TrD virus with a predetermined infectivity titer (PFU/mL), and both positive control calibration wells and internal negative control wells were run on each plate alongside tested samples. For RT-qPCR, we used the forward primer VEEV-F51b (ATGGAGARRGTTCACGTTGAYATCG) and reverse primer VEEV-R159a (GGTCRTTRTCNGTGACCTGCTT), as well as the probe VEEV-p120S-MGB (6FAM - CAGTTTGAGGTAGAAGC – MGBNFQ) for detection. Reactions were run on the LightCycler 480 (Roche, Basel, Switzerland) using the Taqpath™ 1-Step Multiplex Master Mix (Thermo Fisher Scientific, Waltham, MA) according to manufacturer’s recommendations with the following cycling conditions: 25 °C for 2 min (1 cycle), 53 °C for 10 min (1 cycle), 95 °C for 2 min (1 cycle), 95 °C for 3 sec/60 °C for 30 s (45 cycles), and 40 °C for 30 s (1 cycle). A single fluorescence read was taken at the end of each 60 °C step, and a sample was considered positive if the Cq value was less than 40 cycles for a minimum of two replicates. Final viral load concentrations were interpolated from the positive control material standard curve and were expressed in PFU equivalents/mL. Data analysis was performed in GraphPad Prism. All samples collected on days −7, −5, and −3 prior to challenge were negative for virus. For graphing purposes, the negative pre-challenge status was collapsed into time point 0. Additionally, in order to project a viral load of zero on the logarithmic *y* axis, zero values were entered as ones. The lower limit of quantitation of the assay is 100 PFU equivalents/mL.

### Telemetry

Body temperature and activity were continuously monitored by implanted M00 telemetry devices (Data Sciences International, Inc., St. Paul, MN). Devices were surgically implanted by USAMRIID veterinary staff 14 days prior to first vaccination to allow sufficient time for the animals to recover from surgery. Digital data were captured at one sample/second, reduced, and stored in NSS telemetry data files using the Notocord-hem Evolution software platform (Version 4.3.0.77, NOTOCORD Systems, Instem Company, Le Pecq, France). Data in the NSS files were extracted and further reduced into a validated Microsoft Excel workbook for each macaque using Notocord-derived formula add-ins. Data reduction was done in 30 min intervals for temperature and 12 h intervals for animal movement/activity. NSS data files were generated during data acquisition to record 6 days of baseline prior to virus exposure. Significant body temperature elevations and reductions were defined respectively as temperatures >3 SD above and <3 SD below time-matched baseline for longer than 2 h. Fever and hyperpyrexia were defined as body temperatures >1.5 °C and >3.0 °C, respectively, above time-matched baseline for longer than 2 h. Severe hypothermia was defined as body temperature >2.0 °C below time-matched baseline for longer than 2 h.

### Pathology

At the designated end-of-study, a partial necropsy was performed for each animal. As VEEV infection of NHPs has been well-characterized, only a subset of key organs and tissues were collected including the brain, tracheobronchial lymph node, lung, liver, spleen, and vaccination site. Histopathology was performed on the brain, vaccination site, and tracheobronchial lymph node. The lung, liver, and spleen were blocked in paraffin for archiving only. After arrival at the histopathology laboratory, the tissue samples were trimmed, processed, and embedded in paraffin. Sections of the paraffin embedded tissues 5 µm thick were cut for histology. The histology slides were deparaffinized, stained with hematoxylin and eosin (H&E), cover slipped, and labeled. The study pathologist subjectively determined severity scores based on the percentage of tissue examined that was affected by histologic changes: 0-negative; 1-minimal, >0–10%; 2-mild, >10–25%; 3-moderate, >25–50%; and 4-marked, >50–75%.

Immunohistochemistry (IHC) was performed using the Dako Envision system (Dako Agilent Pathology Solutions, Carpinteria, CA). Briefly, after deparaffinization, peroxidase blocking, and antigen retrieval, sections were covered with a rabbit polyclonal anti-alphavirus antibody (#1140, USAMRIID, Frederick, MD) at a dilution of 1:6000 and incubated at room temperature for 45 min. They were rinsed, and the peroxidase-labeled polymer (secondary antibody) was applied for 30 min. Slides were rinsed and a brown chromogenic substrate 3,3’ Diaminobenzidine (DAB) solution (Dako Agilent Pathology Solutions) was applied for 8 min. The substrate-chromogen solution was rinsed off the slides, and slides were counterstained with hematoxylin and rinsed. The sections were dehydrated, cleared with Xyless, and then cover slipped.

### Statistical analysis

Serological data are presented as either the geometric mean titer (GMT) of individual NHPs ± the geometric SD, the group mean of individual NHPs ± the SD, or the group mean of individual NHPs ± the SEM as indicated. Statistical analysis was performed using a Student’s *t-*test or a one-way ANOVA followed by a Tukey’s multiple comparison, or a two-way ANOVA followed by a Tukey’s multiple comparison. Kaplan–Meier survival curve analysis using a log rank test was performed to determine *p*-value significance of vaccinated groups surviving lethal challenge compared to the control group using GraphPad Prism 9 for Windows. Further, log_10_ transformations were applied to VLP end-point ELISA titers using GraphPad 9 software as described above. For the hematology parameters, a Kruskal–Wallis test was performed for each study day to assess overall differences among the three groups. For those days/parameters that were significant by Kruskal–Wallis (Supplementary Table 3), a series of Wilcoxon tests were performed for each pairwise comparison.

### Reporting summary

Further information on research design is available in the Nature Research Reporting Summary linked to this article.

## Supplementary information

Supplementary Material

REPORTING SUMMARY

## Data Availability

The data that support the findings of this study are available from the authors on reasonable request pending approval from all relevant government institutions.
