# Supplementary material for: A DNA vaccine targeting VEE virus delivered by needle-free jet-injection protects macaques against aerosol challenge
Source: NPJ Vaccines. 2022 Apr 22;7:46. doi: 10.1038/s41541-022-00469-x (PMC9033795; doi:10.1038/s41541-022-00469-x)
Supplement: Supplementary file 1 — Supplementary Material [file 41541_2022_469_MOESM1_ESM.pdf]

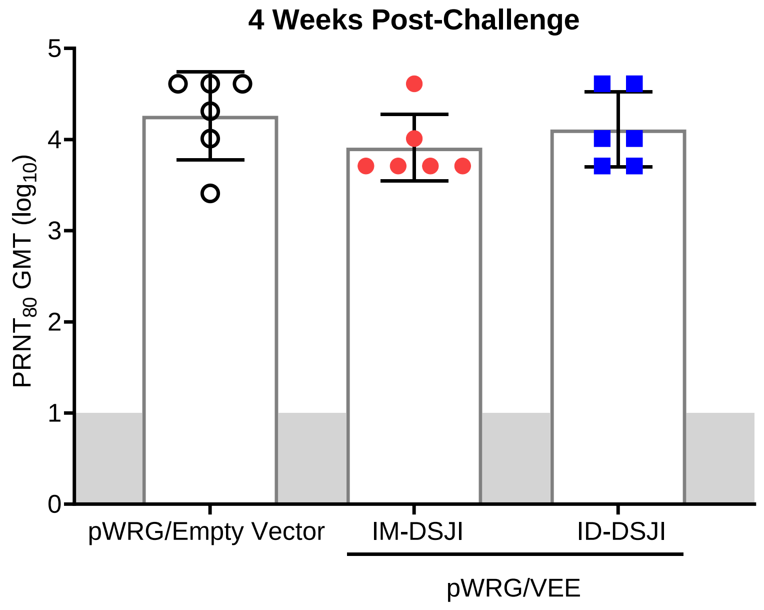

Supplementary Figure 1. Anti-VEEV neutralizing antibodies following aerosol VEEV challenge. $PRNT_{80}$ titers four weeks post VEEV aerosol challenge. The shaded areas represent the assay limit.

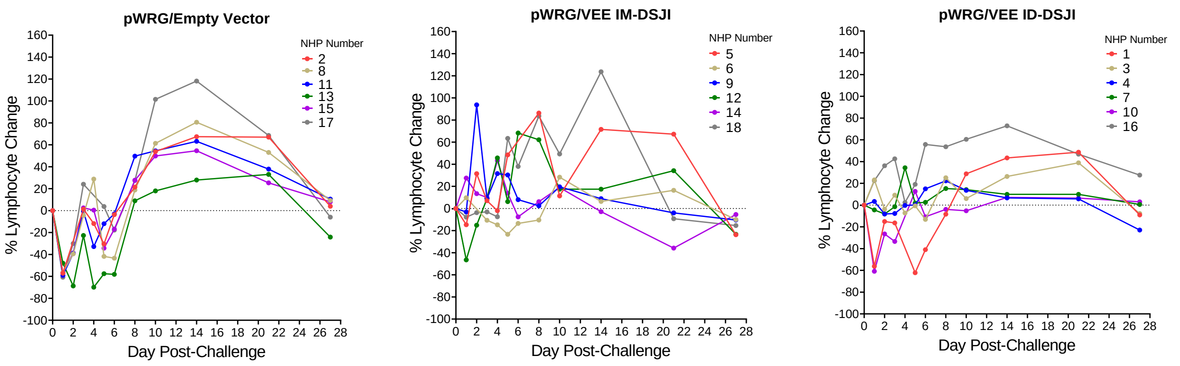

Supplementary Figure 2. pWRG/VEE vaccination protected all IM-DSJI animals from lymphopenia and all but one ID-DSJI animal. Percent change in lymphocyte populations from baseline for individual nonhuman primates (NHP) vaccinated with the indicated vaccine. The dashed line represents the baseline values as determined by the average of three independent measurements on days -7, -5, and -3 relative to challenge.

Supplementary Table 1. Individual nonhuman primate (NHP) inhaled dose

Group	NHP Number	Inhaled Dose (PFU)
pWRG/empty vector	2	6.81E+07
pWRG/empty vector	8	6.62E+07
pWRG/empty vector	11	8.42E+07
pWRG/empty vector	13	7.74E+07
pWRG/empty vector	15	7.04E+07
pWRG/empty vector	17	7.12E+07
pWRG/VEE IM-DSJI	5	7.23E+07
pWRG/VEE IM-DSJI	6	2.76E+06
pWRG/VEE IM-DSJI	9	6.17E+07
pWRG/VEE IM-DSJI	12	7.81E+07
pWRG/VEE IM-DSJI	14	7.32E+07
pWRG/VEE IM-DSJI	18	6.55E+07
pWRG/VEE ID-DSJI	1	6.03E+07
pWRG/VEE ID-DSJI	3	6.97E+07
pWRG/VEE ID-DSJI	4	6.09E+07
pWRG/VEE ID-DSJI	7	6.08E+07
pWRG/VEE ID-DSJI	10	5.58E+07
pWRG/VEE ID-DSJI	16	6.44E+07
	Average Titer (PFU)	6.43E+07

pWRG/empty vector: IM-DSJI 2.0 mg; ID-DSJI 0.4 mg
pWRG/VEE IM-DSJI: 2.0 mg
pWRG/VEE ID-DSJI: 0.4 mg

Supplementary Table 2. Vaccination and immunoassay schedule

DNA Vaccine Group	N	Dose	Route	Procedure Weeks: Vaccination	Procedure Weeks: ELISA & Neutralization	Procedure Weeks: ELISPOT
pWRG/empty vector	6	2.0 mg<br>0.4 mg	IM-DSJI<br>ID-DSJI	0, 4	0, 4, 6, 8	0, 4, 8
pWRG/VEE	6	2.0 mg	IM-DSJI	0, 4	0, 4, 6, 8	0, 4, 8
pWRG/VEE	6	0.4 mg	ID-DSJI	0, 4	0, 4, 6, 8	0, 4, 8

Supplementary Table 3. Summary of significant alterations in hematology parameters

Parameter	Day Post-Challenge	Kruskal-Wallis p-value	Wilcoxon p-value (unadjusted) pWRG/Empty Vector v IM-DSJI	Wilcoxon p-value (unadjusted) pWRG/Empty Vector v ID-DSJI	Wilcoxon p-value (unadjusted) IM-DSJI v ID-DSJI
WBC	3	**0.0314**	0.0927	**0.0305**	1.0000
WBC	4	**0.0493**#	0.0842	0.0709	0.7564
WBC	5	**0.0028**	**0.0172**	**0.0172**	0.3973
Lymphocytes	1	**0.0222**	**0.0172**	0.1208	0.9376
Lymphocytes	2	**0.0053**	**0.0335**	**0.0244**	0.2628
Lymphocytes	5	**0.0222**	**0.0305**	0.2007	0.1564
Lymphocytes	10	**0.0363**#	0.0535	0.0706	0.4862
Neutrophils	5	**0.0102**	**0.0305**	**0.0305**	0.5864
Basophils	3	**0.0311**	0.0922	**0.0300**	1.0000
Basophils	4	**0.0133**	**0.0334**	**0.0484**	0.9174
Eosinophils	3	**0.0192**	**0.0404**	**0.0404**	0.9376

Values in bold are considered significant ($p<0.05$)

Pairwise comparison by Wilcoxon test were suggestive of a difference between groups, but were not statistically conclusive
