# Supplementary material for: A DNA vaccine targeting VEE virus delivered by needle-free jet-injection protects macaques against aerosol challenge
Source: NPJ Vaccines. 2022 Apr 22;7:46. doi: 10.1038/s41541-022-00469-x (PMC9033795; doi:10.1038/s41541-022-00469-x)
Supplement: Supplementary file 2 — REPORTING SUMMARY [file 41541_2022_469_MOESM2_ESM.pdf]

nature portfolio

Corresponding author(s): Hooper

Last updated by author(s): Jan 19, 2022

Reporting Summary

Nature Portfolio wishes to improve the reproducibility of the work that we publish. This form provides structure for consistency and transparency in reporting. For further information on Nature Portfolio policies, see our Editorial Policies and the Editorial Policy Checklist.

Statistics

For all statistical analyses, confirm that the following items are present in the figure legend, table legend, main text, or Methods section.

n/a	Confirmed	
☐	☒	The exact sample size (*n*) for each experimental group/condition, given as a discrete number and unit of measurement
☐	☒	A statement on whether measurements were taken from distinct samples or whether the same sample was measured repeatedly
☐	☒	The statistical test(s) used AND whether they are one- or two-sided<br>*Only common tests should be described solely by name; describe more complex techniques in the Methods section.*
☐	☒	A description of all covariates tested
☐	☒	A description of any assumptions or corrections, such as tests of normality and adjustment for multiple comparisons
☐	☒	A full description of the statistical parameters including central tendency (e.g. means) or other basic estimates (e.g. regression coefficient) AND variation (e.g. standard deviation) or associated estimates of uncertainty (e.g. confidence intervals)
☐	☒	For null hypothesis testing, the test statistic (e.g. *F*, *t*, *r*) with confidence intervals, effect sizes, degrees of freedom and *P* value noted<br>*Give P values as exact values whenever suitable.*
☒	☐	For Bayesian analysis, information on the choice of priors and Markov chain Monte Carlo settings
☒	☐	For hierarchical and complex designs, identification of the appropriate level for tests and full reporting of outcomes
☐	☒	Estimates of effect sizes (e.g. Cohen's *d*, Pearson's *r*), indicating how they were calculated

Our web collection on statistics for biologists contains articles on many of the points above.

Software and code

Policy information about availability of computer code

Data collection	N/A
Data analysis	GraphPad Prism 9

For manuscripts utilizing custom algorithms or software that are central to the research but not yet described in published literature, software must be made available to editors and reviewers. We strongly encourage code deposition in a community repository (e.g. GitHub). See the Nature Portfolio guidelines for submitting code & software for further information.

Data

Policy information about availability of data

All manuscripts must include a data availability statement. This statement should provide the following information, where applicable:

- Accession codes, unique identifiers, or web links for publicly available datasets
- A description of any restrictions on data availability
- For clinical datasets or third party data, please ensure that the statement adheres to our policy

The data that support the findings of this study are available from the authors on reasonable request pending approval from all relevant government institutions.

March 2021

Field-specific reporting

Please select the one below that is the best fit for your research. If you are not sure, read the appropriate sections before making your selection.

☒ Life sciences ☐ Behavioural & social sciences ☐ Ecological, evolutionary & environmental sciences

For a reference copy of the document with all sections, see nature.com/documents/nr-reporting-summary-flat.pdf

Life sciences study design

All studies must disclose on these points even when the disclosure is negative.

Sample size	Using a onetailed Fisher's exact test to compare survival rates between the control and treatment groups, this experiment requires a sample size of 6 animals per group for adequate (>80%) power. This sample size would allow the experimenter to detect a minimum efficacy rate of 100% (6/6 surviving) in the treatment group compared to ≤17% (1/6 surviving) in the control group at a 95% confidence level.
Data exclusions	N/A
Replication	N/A
Randomization	Animals were randomized into groups by statistician.
Blinding	Technicians performing immunoassays and animal checks were blinded to group identity.

Reporting for specific materials, systems and methods

We require information from authors about some types of materials, experimental systems and methods used in many studies. Here, indicate whether each material, system or method listed is relevant to your study. If you are not sure if a list item applies to your research, read the appropriate section before selecting a response.

Materials & experimental systems

n/a	Involved in the study	
☐	☒	Antibodies
☐	☒	Eukaryotic cell lines
☒	☐	Palaeontology and archaeology
☐	☒	Animals and other organisms
☒	☐	Human research participants
☒	☐	Clinical data
☒	☐	Dual use research of concern

Methods

n/a	Involved in the study	
☒	☐	ChIP-seq
☒	☐	Flow cytometry
☒	☐	MRI-based neuroimaging

Antibodies

Antibodies used	anti-CD3 monoclonal antibody from Mabtech, Cincinnati, OH
Validation	Commercial product. "The included anti-CD3 mAb can be used to induce cytokine secretion and is dependent on number, nature and viability of accessory cells. The antibody cross-reacts with CD3 on cynomolgus and rhesus macaque T cells."

Eukaryotic cell lines

Policy information about cell lines

Cell line source(s)	Vero 76 (ATCC, Manassas, VA)
Authentication	none
Mycoplasma contamination	none
Commonly misidentified lines (See ICLAC register)	*Name any commonly misidentified cell lines used in the study and provide a rationale for their use.*

Animals and other organisms

Policy information about studies involving animals; ARRIVE guidelines recommended for reporting animal research

Laboratory animals	18 healthy adult male and female cynomolgus macaques >4 kg
Wild animals	N/A
Field-collected samples	N/A
Ethics oversight	Research was conducted under an animal protocol approved by the IACUC at the United States Army Medical Research Institute of Infectious Diseases (USAMRIID).

Note that full information on the approval of the study protocol must also be provided in the manuscript.
